# Aims of the Journal

**Published:** 1881-10

**Authors:** 


### AIMS OF THE JOURNAL.

THE first and immediate aim of the good and great physician, is to restore his patient to health in the shortest time, with the smallest amount of medicine, and with the least discomfort practicable; when this is accomplished, he has a more elevated ambition; an object nobler and still more humane presses upon his attention—*the prevention of all disease.* This good time may not come, in the broadest acceptation of the terms; but for generations past, medical men have so steadily labored in that direction, and do still labor, that the average duration of human life has been constantly raised.

"In the latter part of the sixteenth century," according to Professor Joseph R. Buchanan, of Cincinnati, "one half of all who were born, died under five years of age, and the average longevity of the whole population was but eighteen years.

"In the seventeenth century, one half of the population lived over twenty-seven years. In the latter forty years, one half exceeded thirty-two years of age.

"At the beginning of the present century, one half exceeded forty years of age; and from 1838 to 1845 one half exceeded forty-three years—that is to say, in the sixteenth century one half of all who were born lived only five years, while in the present century, which is the nineteenth, one half of all who are born live to the age of forty-three years." To accomplish such magnificent results, educated and honorable physicians have devoted their energies with increasing success for the last three hundred years; and to them the world owes a debt of gratitude which cannot be easily computed. And thus they labor still, hoping for yet higher results from the diffusion of general knowledge as to the best methods of preserving health, by teachings as to the laws of our being in relation to air, exercise, food, sleep, personal habits, clothing, the locality and construction of houses, and the management of infants. And aided by the ever-widening influences of the principles of the christian system, which, by inculcating, as one of its cardinal elements, "*temperance* in all things," strikes at the very root of disease, we may reasonably hope that, when the true knowledge covers the earth as the waters cover the face of the great deep, the ordinary average of human life will be the full three-score years and ten, or even four-score years, which will then *not* be years of labor and sorrow.

The world would hail it as a glad event, if physicians could be so educated as to cure all disease; but it would more largely add to its happiness if all could be so well instructed, as to the first symptoms of every ailment, as to be able at once to arrest its progress, and thus no physician be needed to cure; and yet any one must know, that if men could be so taught to live that disease would not be possible, half the sufferings of humanity would be annihilated. *And for this I labor.*

☞ *To teach men how to avoid disease* was the idea which first prompted the determination to publish this periodical, and the only pledge or promise I can give is, that whatever is herein published, will be designed more or less directly, in my opinion, to tend in that direction.

